# Finite element analyses of three minimally invasive fixation techniques for treating Sanders type II intra-articular calcaneal fractures

**DOI:** 10.1186/s13018-023-04244-z

**Published:** 2023-11-28

**Authors:** Guoxun Song, Wenqi Gu, Zhongmin Shi, Xueqian Li, Shaoling Fu, Xiaowei Yu, Facheng Song

**Affiliations:** 1 Department of Orthopaedics, Shanghai Jiaotong University Affiliated Sixth People’s Hospital, Shanghai, 200233 People’s Republic of China; 2 National Key Laboratory for Manufacturing Systems Engineering, Xian Jiaotong University, Xi’an, 710054 Shanxi Province People’s Republic of China

**Keywords:** Finite element, Fracture, Intra-articular calcaneal fracture, Locking plate, Minimally invasive, Screw fixation

## Abstract

**Background and objective:**

Calcaneal Sanders type II or III fractures are highly disabling with significant burden. Surgical treatment modalities include open reduction and internal fixation (ORIF) techniques and a variety of minimally invasive surgical (MIS) approaches. ORIF techniques are associated with complications and traditional MIS techniques need extensive intraoperative fluoroscopic procedures. The present study aims to investigate the effects of three different minimally invasive internal fixation (MIIF) techniques used to treat Sanders type II intra-articular calcaneal fractures using finite element analyses.

**Methods:**

A 64-row spiral computed tomography scan was used to observe the calcaneus of a healthy adult. The scanning data were imported into Mimics in a DICOM format. Using a new model of a Sanders type II-B intra-articular calcaneal fracture, three minimally invasive techniques were simulated. Technique A involved fixation using an isolated minimally invasive locking plate; Technique B used a minimally invasive locking plate with one medial support screw; and Technique C simulated a screw fixation technique using four 4.0-mm screws. After simulating a 640-N load on the subtalar facet, the maximum displacement and von Mises stress of fragments and implants were recorded to evaluate the biomechanical stability of different fixation techniques using finite element analyses.

**Results:**

After stress loading, the maximum displacements of the fragments and implants were located at the sustentaculum tali and the tip of sustentaculum tali screw, respectively, in the three techniques; however, among the three techniques, Technique B had better results for displacement of both. The maximum von Mises stress on the fragments was < 56 Mpa, and stress on the implants using the three techniques was less than the yield strength**,** with Technique C having the least stress.

**Conclusion:**

All three techniques were successful in providing a stable fixation for Sanders type II intra-articular calcaneal fractures, while the minimally invasive calcaneal locking plate with medial support screw fixation approach exhibited greater stability, leading to improved enhancement for the facet fragment; however, screw fixation dispersed the stress more effectively than the other two techniques.

## Introduction

Calcaneal fractures are one of the most challenging and divisive injuries to treat [1]. They are uncommon but serious injuries often caused by trauma. They account for approximately 1–2% of all adult fractures, and displaced intra-articular fractures account for 60–75% of calcaneal fractures [2]. These fractures are highly disabling so that approximately one-fifth of patients with intra-articular calcaneal fractures are unable to return to work for 1 year resulting in significant economic and social burden [3]. Different techniques including operative and non-operative management, impulse compression, and no impulse compression techniques have been developed for treatment of calcaneal fractures. However, there is no consensus on the optimal surgical management for these calcaneal fractures. The main reasons for the controversy are high frequency of post-surgery complications and the correlation between anatomical restoration and outcome (function, quality of life) is not yet proven [4]. Over the last decades, significant advances have been achieved in operative management of displaced intra-articular calcaneal fractures; however, they are often associated with different complications such as long-term sequelae, reduced quality of life, and permanent disability. Fractures with significant lateral wall displacement with high risk of impingements often need surgical management that should be individualized and patient-specific. Extensile lateral approaches are associated with wound healing impairments and infections affecting approximately 20% of patients. Minimally invasive surgery and sinus tarsi approach with comparable outcomes, yet fewer wound complications, but the available studies are mostly of low to moderate quality. For displaced intra-articular calcaneal fractures, late subtalar joint arthrodesis is often required. This procedure should be technically more feasible resulting in better foot function through which the calcaneus shape is anatomically restored with surgery in the acute phase [5]. Closed reduction with percutaneous pinning is a minimally invasive surgical (MIS) technique and reportedly associated with fewer wound and infection complications compared with open reduction. The traditional and classic MIS technique for treating calcaneal fractures comprises of fracture reduction, temporary fixation, and then internal fixation along with an implant. Traditional techniques of minimally invasive internal fixation (MIIF) for calcaneal fractures demand time-consuming intraoperative fluoroscopic procedure, though the fracture healing is not ideal. Therefore, it is necessary to develop new MIS procedures based on efficient modeling and digital surgical simulation to design effective surgical procedure and build a patient-specific instrument for calcaneal fracture during the surgical procedure.

Although a variety of surgical approaches have been used to treat displaced intra-articular calcaneal fractures, the extensile lateral approach has been the most common technique over the past 30 years. However, a reportedly ~ 25–30% of soft tissue complications remains a great challenge when using this technique [6, 7]. With the development of MIS techniques, the rate of soft tissue complications has significantly decreased in recent years [8]. Closed reduction and internal fixation is a classic MIS technique that has been modified in recent years for use in treatment of intra-articular calcaneal fractures with an acceptable clinical outcome [9]; however, because the articular surface was not readily observed, the malreduction in facet fragments and post-traumatic arthritis remained high risk. The limited open reduction and internal fixation (ORIF) through a sinus tarsi or other modified minimally invasive incisions have been the mainstream of the MIS technique in recent years [10–15]. The subtalar joint can be clearly exposed using the sinus tarsi approach, and the reduction and internal fixation can be performed under direct vision, which could effectively decrease the complication rate caused by malreduction. The classic fixation method for limited ORIF is screw fixation using at least four screws; however, with the development of newly designed implants, another advantage of this technique is that the fracture can be stabilized using a minimally invasive locking plate. Arthroscopic-assisted reduction and percutaneous screw fixation have become increasingly popular [16–18], and the subtalar fragment can be reduced and stabilized with arthroscopy followed by percutaneous screw fixation, which might be more cost effective than plate fixation; however, the arthroscopic technique is highly demanding with a steep learning curve. Although different fixation techniques can be applied, the one that achieves better stability remains controversial. Therefore, the present study was aimed to investigate the biomechanical effects of three different MIIF techniques used to treat Sanders type II intra-articular calcaneal fractures. We hypothesized that the MIS calcaneal locking plate with medial support screw fixation would provide better biomechanical stability for the fracture.

## Materials and methods

### Data collection

A 28-year-old male patient weighing 64 kg and height of 1.71 m without any history of foot and ankle trauma, deformity, or other diseases was enrolled in the study. The 64-row computed tomography (CT) scanner (Toshiba Aquilion, Japan) was used to scan and provide three-dimensional (3D) reconstruction of the calcaneus in a 1-mm-thick scanning layer. The computed tomography (CT) images in the matrix of 512 × 512 (DICOM format) were obtained after scanning.

### Model establishment

The models used were developed using the protocols listed below.

#### Establishment of a finite element model of a Sanders type II intra-articular calcaneal fracture

**Fig. 1:**
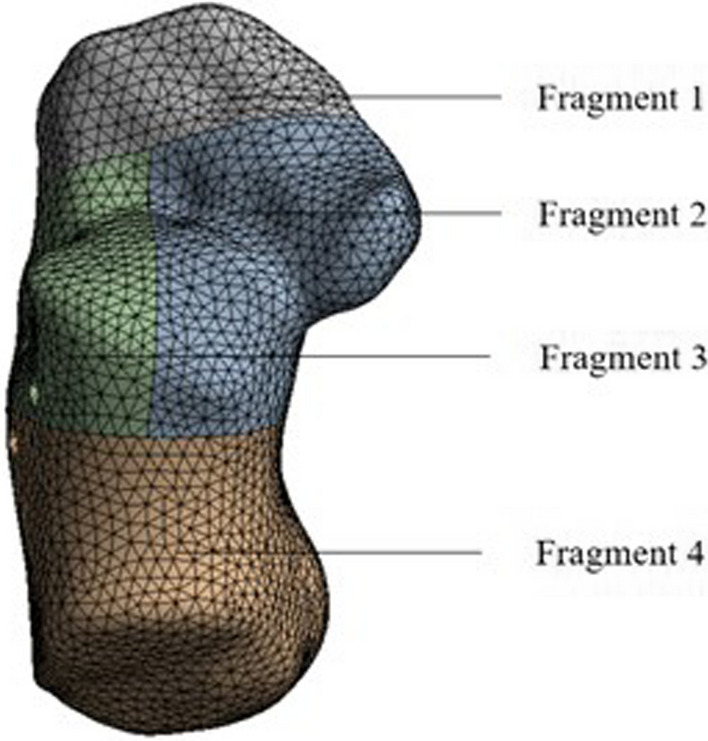
Model of Sanders type II-B intra-articular calcaneal fracture

Based on the selected CT images, the 3D model of calcaneus was established using threshold segmentation. The CT images were imported into MIMICS, and the bony structure was extracted to obtain a complete set of calcaneus data, which were then exported in STL format. In Geomagic Studio, the calcaneus data were further processed for smoothing, slacking and feature elimination. According to the typical pattern of a Sanders type II-B calcaneal fracture, the calcaneus was divided into four fragments as follows to generate a 3D geometric model: anterior fragment (fragment 1), medial fragment including medial facet of subtalar joint and sustentaculum tali (fragment 2), lateral fragment with lateral facet of subtalar joint (fragment 3), and the calcaneal tuberosity fragment (fragment 4). The fragments were processed for curved surface to generate a nonuniform rational basis spline (NURBS) surface. Then, the model of the Sanders type II-B intra-articular calcaneal fracture (Fig. 1) was imported into Geomagic DesignX for fixation simulation.

##### Establishment of geometric model of minimally invasive calcaneal locking plate and cannulated screw

The data on the minimally invasive calcaneal locking plate (Acumed, Hillsboro, OR, USA) were scanned to build geometric models through inverse processing approach. The screw thread was simplified, and the forward geometric models of the screws were established. The geometric parameters of the implants were imported with the screw of the plate system set as a φ3.5-mm rod, and other screws as φ4.0-mm rods. Then, the coordinate system was set up, and the geometrical parameters were imported.

##### Fixation techniques

**Fig. 2:**
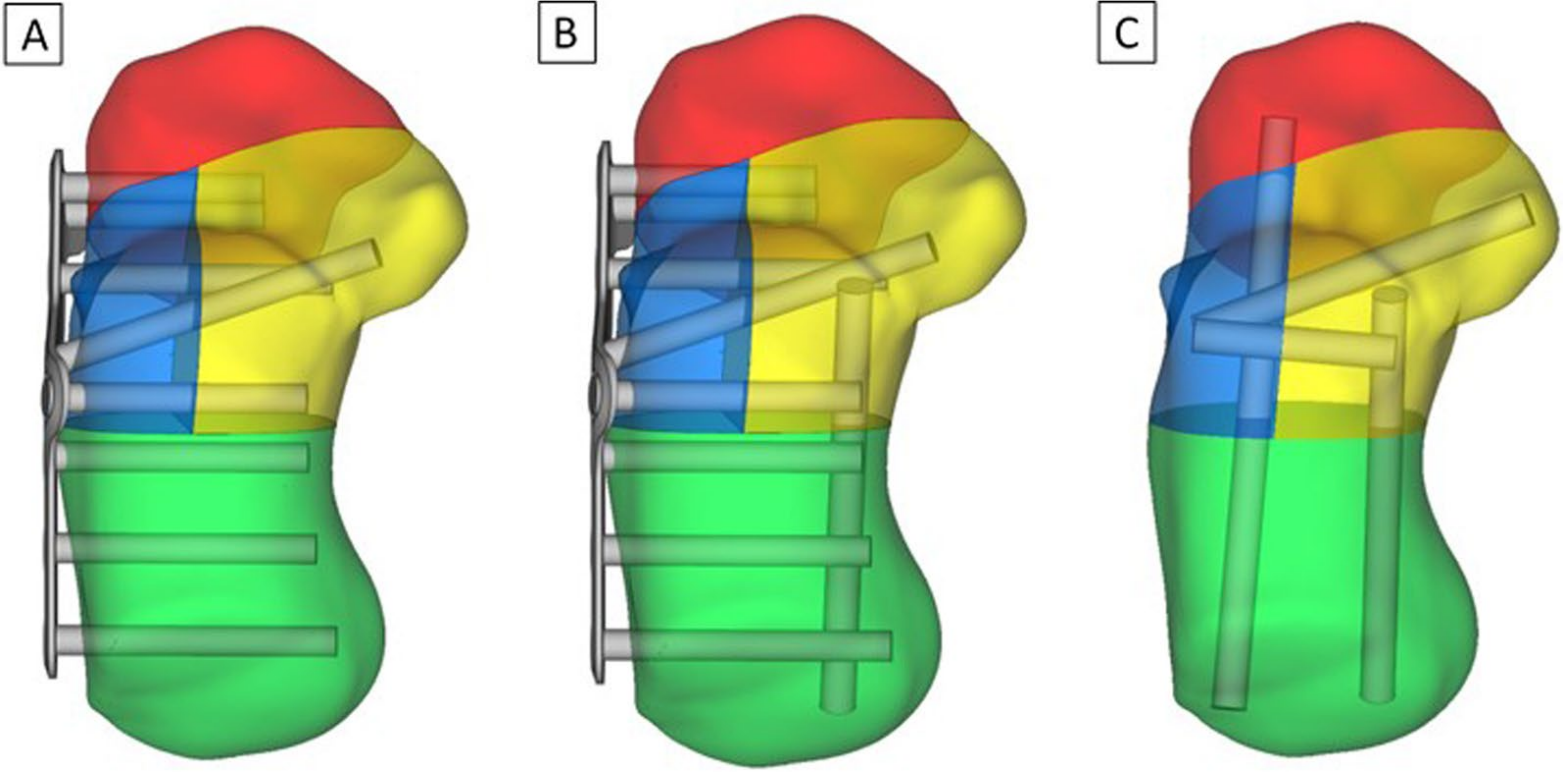
Three minimally invasive surgical fixation techniques. **A** Technique A using a minimally invasive calcaneal locking-plate fixation; **B** Technique B using a minimally invasive calcaneal locking plate with a medial support fixation; and **C** Technique C using screws fixation

In the Geomagic DesignX tool, three different minimally invasive fixation techniques were simulated. The three techniques, named A, B, and C are described below, respectively. Technique A: The fracture was stabilized using only a minimally invasive calcaneal locking plate with two screws in the anterior fragment, three screws in the lateral and medial fragment with one screw fixation for the sustentaculum tali, and three screws fixation in the tuberosity. Technique B: The fracture was fixed with a minimally invasive calcaneal locking plate using the same method as Technique A, but with the addition of a medial support screw from the medial tuberosity to the medial fragment underneath the medial facet. Technique C: The fracture was simulated as a screw fixation with one from the lateral tuberosity to the anterior fragment; one from the medial tuberosity to the medial fragment underneath the medial facet and two from the lateral fragment to the medial fragment, one of which was fixed in the sustentaculum tali (Fig. 2A–C).

### Finite element calculation

#### Material property

The fixation model was imported into ANSYS (WINDOWS, ver. 19.2) finite element analysis software. The material properties are defined according to the parameters given in Table 1 with the bony structure which was set as a linear model of isotropic homogeneity.

**Table 1:** Material parameters for the models in the study

Model	Elastic modulus (Mpa)	Poisson’s ratio
Implant	110,000	0.3
Cortical bone	7300	0.3
Cancellous bone	620	0.3

#### Boundary conditions and loads

The friction coefficient between the fragments was set as 0.2, while the friction coefficient between the fragments and implant was set as 0.5. The calcaneocuboid facet and the contact area between the calcaneus and ground was sustained. It has been reported that stress on the Achilles tendon sustains about 50% of the force of the foot [19]. Using the candidate’s weight (64 kg) as a reference, one foot sustained 320 N and the Achilles tendon sustained 160 N of pressure while standing; therefore, 160 N was loaded in the direction of the Achilles tendon to simulate the maximum tensile stress that the calcaneus sustained.

The stress loading in the present study comprised two parts—the subtalar facet sustained a 640-N load, while an upward load of 160 N was sustained on the Achilles tendon insertion of calcaneal tuberosity. The mesh generation was chosen in the directory tree, the mesh of fracture model was set as 2 mm, while the mesh of implant was set as 1 mm, and then the tetrahedral mesh was established. The final finite element analysis was resolved, and the displacement and stress of fragments and implants were recorded.

The criteria considered to determine the results were as follows [20]: (1) screw loosening and bone resorption occurred when compressive strain was > 56 MPa per module on the calcaneus around the screws; and (2) stress > 600 MPa implied a plate breakage, while that > 450 Mpa (yield strength) indicated a risk of implant failure. For further evaluation on the accuracy and precision of the finite element models, we used the same parameters of materials and stress loading and compared the model’s outcomes for displacement and von Mises stress of fragments and implants. The outcomes for different repeated calculations were the same. Therefore, we did not replicate the finite element analysis.

## Results

### Maximum displacement of fragments in the three fixation techniques

**Fig. 3:**
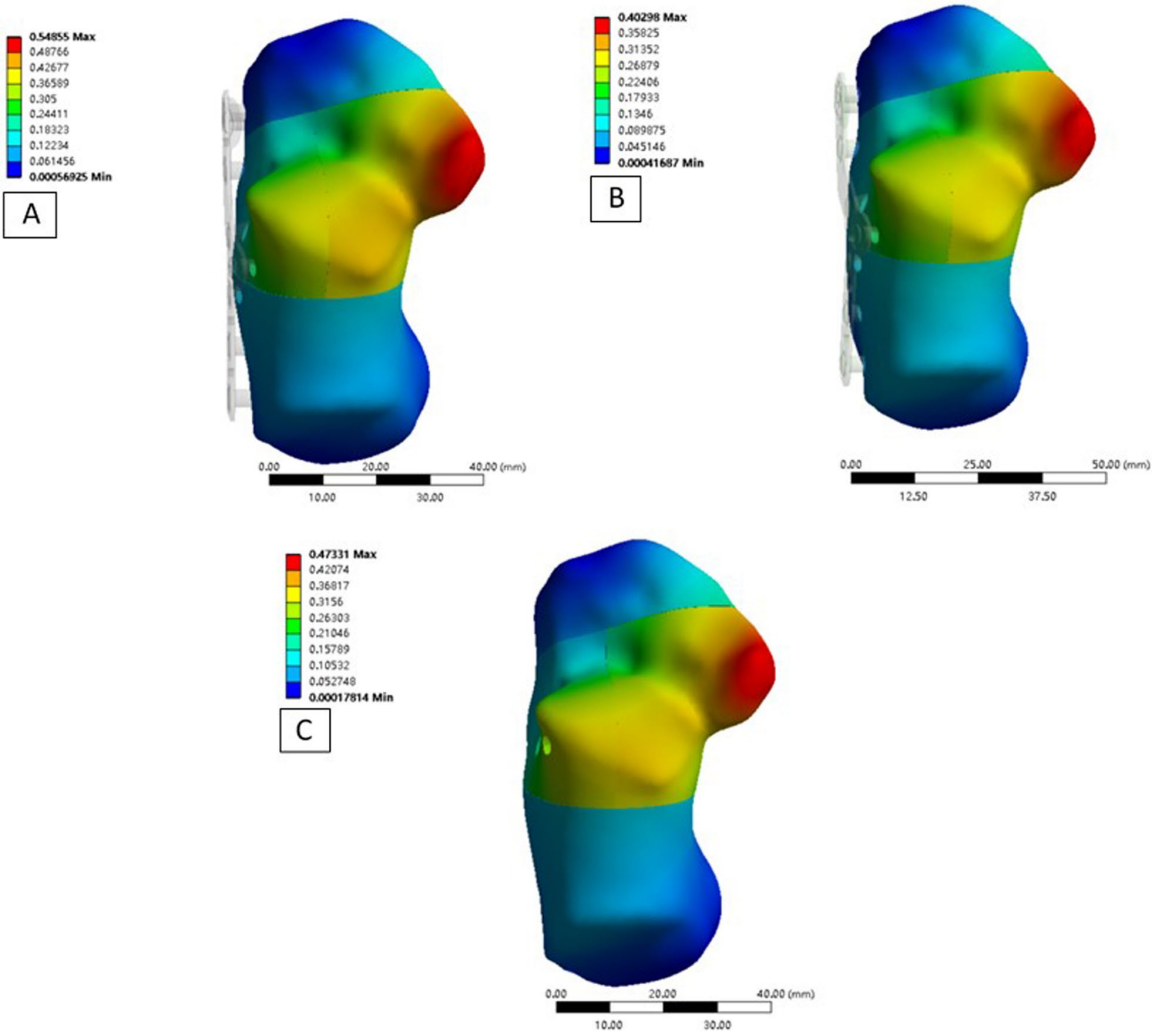
Maximum displacement of fragments. **A** Technique A; **B** Technique B; **C** Technique C

The maximum displacement of the fragments from the three fixation methods was as follows: (1) Technique A: fragment 1 was 0.20 mm, fragment 2 was 0.55 mm, fragment 3 was 0.40 mm, and fragment 4 was 0.15 mm; (2) Technique B: fragment 1 was 0.18 mm, fragment 2 was 0.40 mm, fragment 3 was 0.29 mm, and fragment 4 was 0.12 mm; (3) Technique 3: fragment 1 was 0.19 mm, fragment 2 was 0.47 mm, fragment 3 was 0.36 mm, and fragment 4 was 0.15 mm. The maximum displacement of all fragments was at the medial facet fragment (Figs. 3, 5). Among the three techniques, Technique B resulted in better fragment stability.

### Maximum displacement of implants from three fixation techniques

**Fig. 4:**
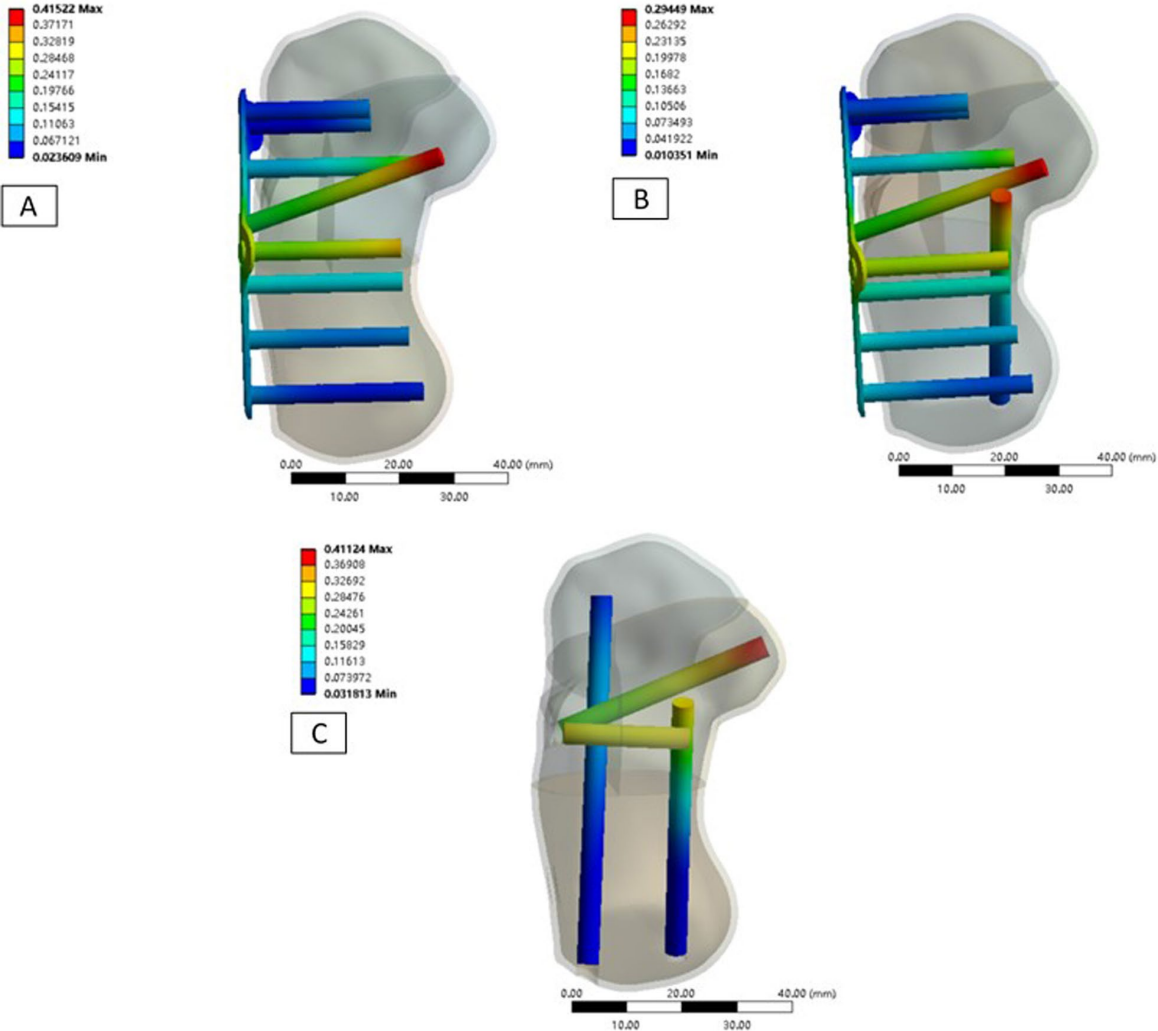
Maximum of displacement of implants. **A** Technique A, **B** Technique B, and **C** Technique C

**Fig. 5:**
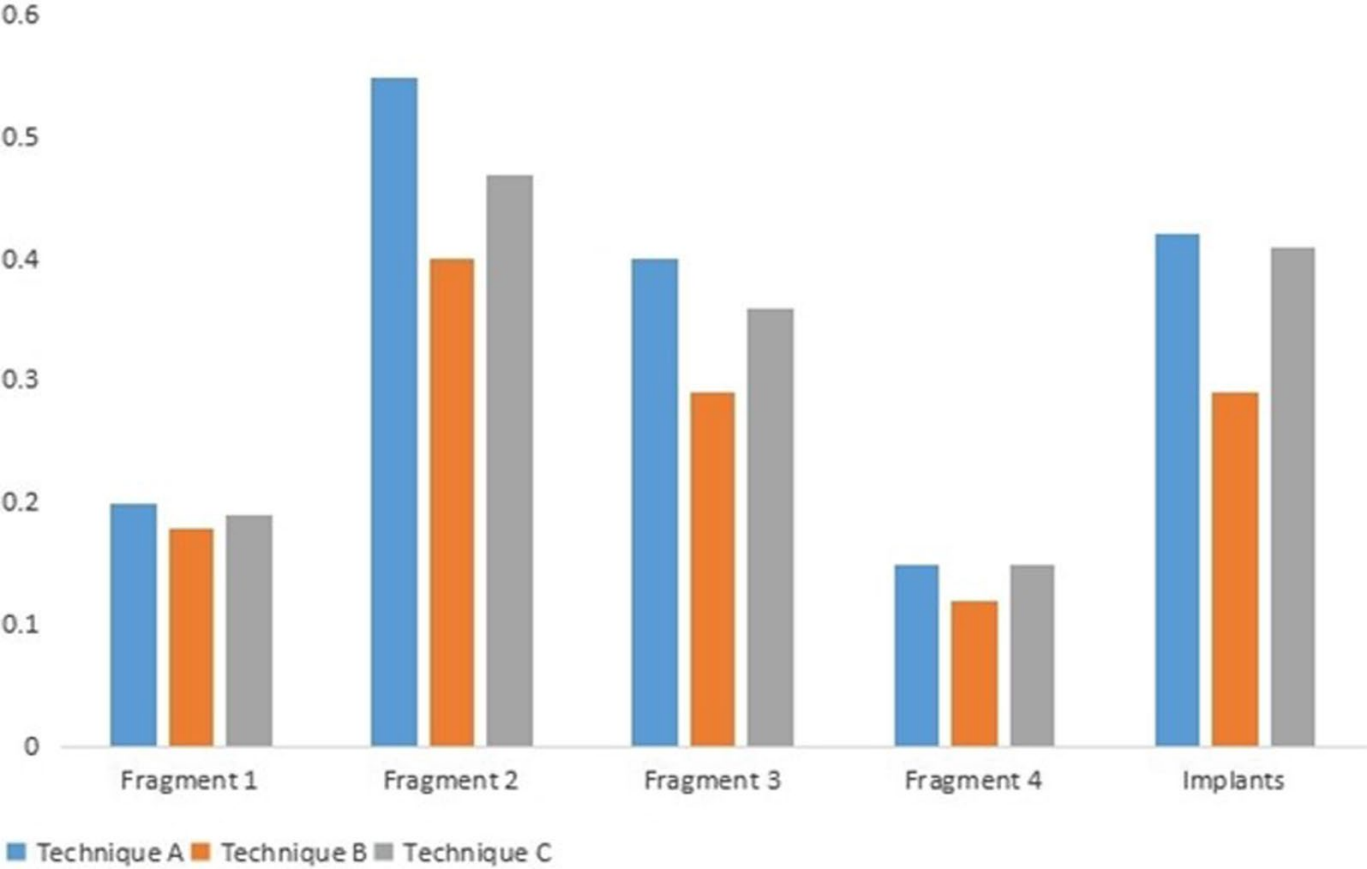
Comparisons of maximum displacement (mm) of fragments and implants between the three techniques

The maximum displacement of the implants from the three fixation techniques was as follows: (1) Technique A: 0.42 mm, (2) Technique B: 0.29 mm, and (3) Technique C: 0.41 mm. Displacement was located at the tip of sustentaculum tali screw (Figs. 4, 5). However, there was less displacement with Technique B than with the other two techniques.

### Maximum von Mises stress on the fragments from the three fixation techniques

**Fig. 6:**
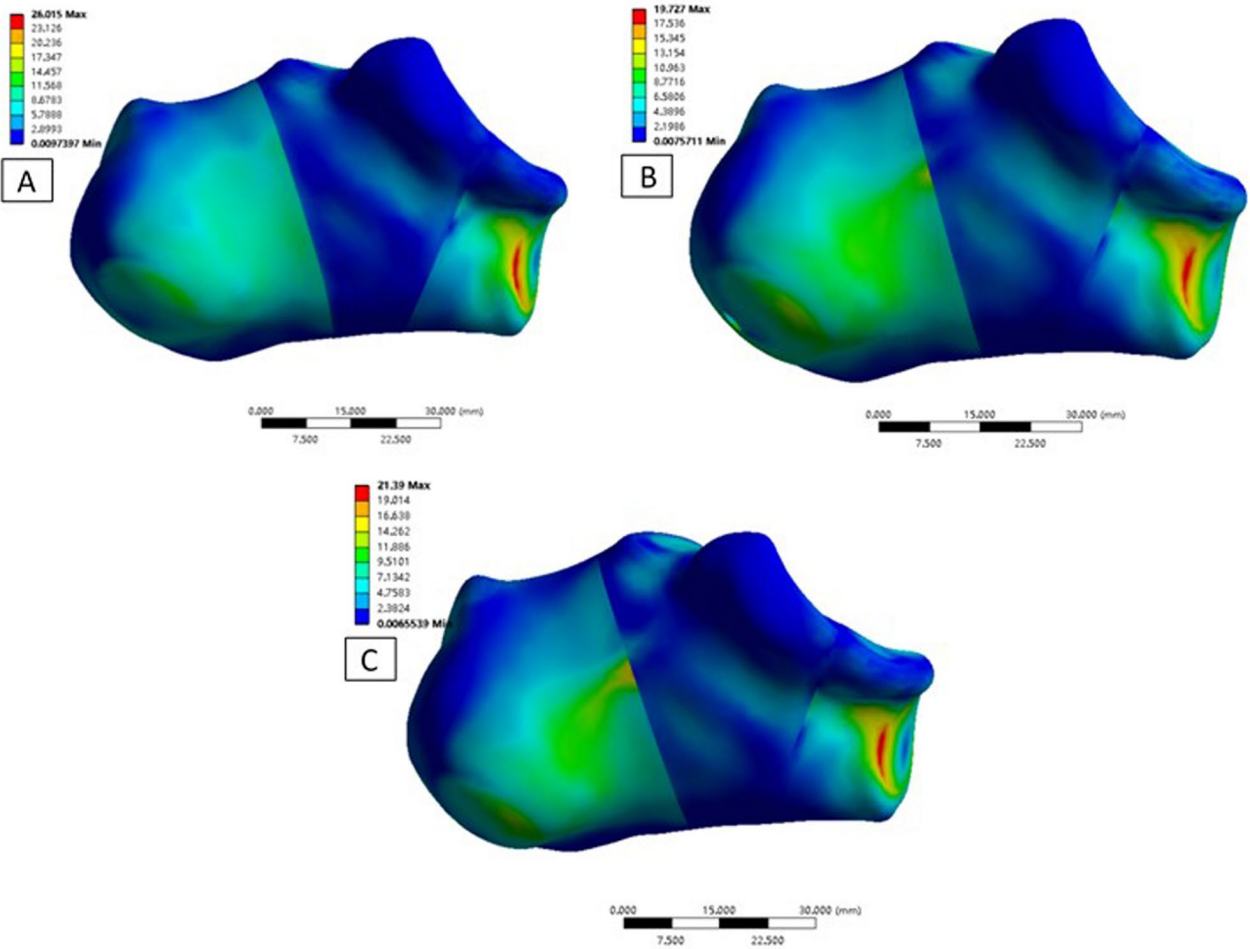
Nephogram of maximum Von Mises stress in the studied fragments. **A** Technique A, **B** Technique B, and **C** Technique C

**Fig. 7:**
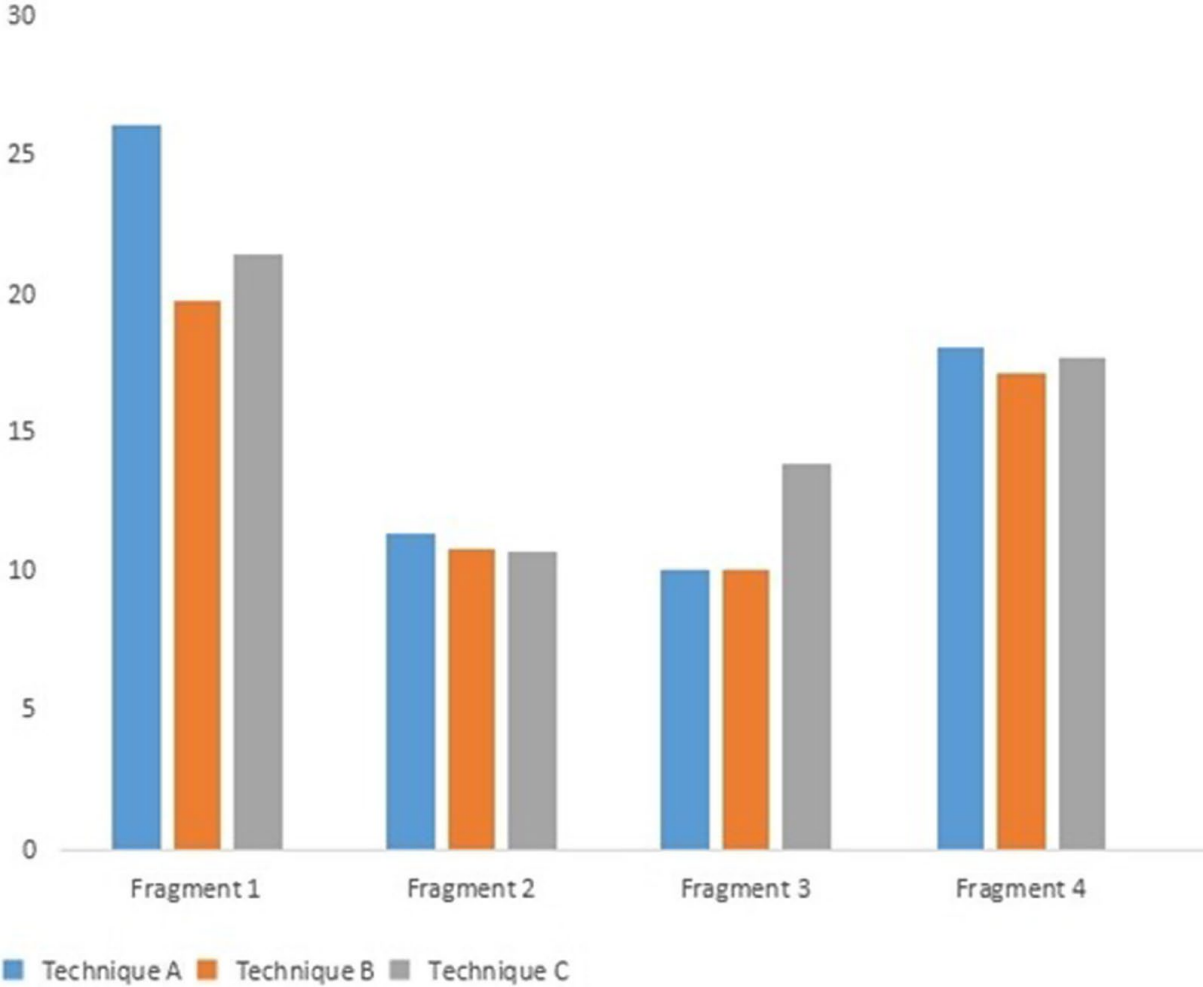
Comparisons of maximum Von Mises stress (Mpa) in the studied fragments between the three techniques

After stress loading, the maximum von Mises stress on the fragments from the three techniques was as follows: (1) Technique A: fragment 1 was 26.02 MPa, fragment 2 was 11.42 MPa, fragment 3 was 10.03 MPa, and fragment 4 was 18.07 MPa; (2) Technique B: fragment 1 was 19.73 MPa, fragment 2 was 10.84 MPa, fragment 3 was 10.11 MPa, and fragment 4 was 17.10 MPa, and (3) Technique C: fragment 1 was 21.39 MPa, fragment 2 was 10.75 MPa, fragment 3 was 13.84 MPa, and fragment 4 was 17.69 MPa. The maximum stress with all three techniques was on the anterior fragment (Figs. 6, 7).

### Maximum von Mises stress on the implants from the three fixation techniques

**Fig. 8:**
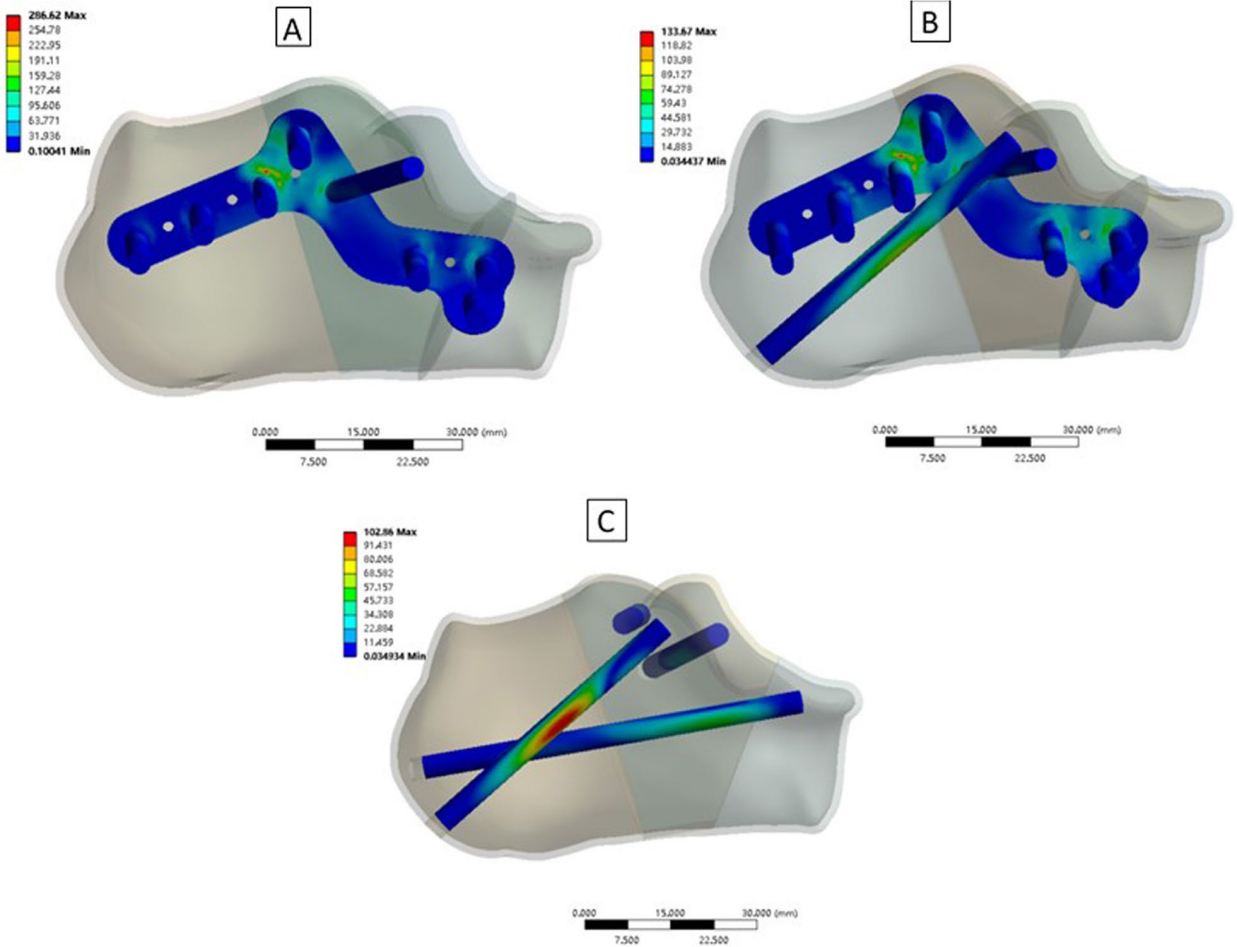
Maximum Von Mises stress on implants. **A** Technique A, **B** Technique B, and **C** Technique C

The maximum von Mises stress on the implants from the three fixation techniques was as follows: (1) Technique A: 286.62 MPa, (2) Technique B: 133.67 MPa; and (3) Technique C: 102.86 MPa. Stress was better dispersed using Technique C (Fig. 8).

## Discussion

Open reduction and internal fixation or arthrodesis for calcaneus via a large incision may lead to various complications, including infections, neurovascular damage and poor wound healing, especially in cases of the high-risk foot with severe soft tissue injuries [21]. Therefore, over the last decades, MIS techniques have gained good popularity among physicians and surgeons for treatment of different intra-articular calcaneal fractures. Limited ORIF, which has remained the mainstream method to treat Sanders type II calcaneal fractures owing to its potential advantages of much shorter surgery duration, theoretically less soft tissue complications, and visibility of the articular surface. We reported a satisfactory clinical outcome using a 2.7-mm mini-fragment locking plate and longitudinal screw fixation [11] and considered that this technique exhibits several advantages. First, the locking plate achieves a better fixation for comminuted or osteoporosis fractures. Second, the rafting technique with a mini-fragment locking plate creates a stable support for the subtalar fragments. Third, the longitudinal screws provide stabilization for tuberosity to prevent re-displacement and varus deformity. In particular, the medial screw might enhance the stability of medial column. The finite element analyses also demonstrated better results for this minimally invasive fixation technique [20]; however, some deficiencies using this technique have been identified. First, the mini-fragment locking plate is usually applied for metacarpal or metatarsal fractures, which is not as useful for the calcaneal fractures; therefore, it would not provide an entire fixation for all fragments, especially for the tuberosity fragment. Second, the plate must be contoured before being implanted, and the biomechanical strength might thus be compromised. The newly designed minimally invasive calcaneal locking plate could resolve these problems. The anatomical design provides a better fit to the calcaneus, and the 3.5-mm system could result in a more stable biomechanical effect than the 2.7-mm plates. Furthermore, the plate has locking screw holes especially for tuberosity; therefore, the necessity of a medial support screw is controversial and worth further discussion. Screw fixation is an economic alternative and constituted of a classic fixation method for almost all MIS techniques for intra-articular calcaneal fractures including limited ORIF, arthroscopic-assisted reduction, and closed reduction percutaneous fixation. Biz et al. compared the clinical and radiological outcomes of ORIF and percutaneous screw fixation in the treatment of displaced intra-articular calcaneal fractures (*n* = 104) [21] and reported that ORIF technique, despite its risk of complications, exhibited better outcomes than percutaneous screw fixation method, though the difference was not statistically significant for intra-articular calcaneal fractures which was inferior to the ORIF, despite without statistical significance. Therefore, whether the screw fixation could result in reliable stabilization also should be determined by basic research. Based on the above, we attempted to investigate the biomechanical effect of different minimally invasive fixation techniques to treat a Sanders type II intra-articular calcaneal fracture, and we hypothesized that the method of minimally invasive calcaneal locking plate with medial support screw fixation could provide a better biomechanical stability for the fracture.

In last decade finite element analyses have been extensively used in evaluation, optimization, and developing new methods for effective management of fractures, especially for comparison of different fixation techniques for calcaneal fractures [22]. Findings of different modeling and experimental studies have shown that the finite element techniques could be used in bone and implant geometries, material properties, meshing, interactions, and loads and boundary conditions. The main applications of these models are predicting implant stress, bone strain surrounding screws, or interfragmentary displacements [23, 24]. However, a major issue on these models is validated because most models are not rigorously validated.

With refined modeling methods, improved validation efforts, and large-scale systematic analyses, finite element analysis is poised to advance the understanding of fracture fixation failure, enable optimization of implant designs, and improve surgical guidance.

Previous studies have developed several intra-articular calcaneal fracture models [25–28]. In the present study, we simulated a Sanders type II-B intra-articular calcaneal fracture model because this is one of the most common types of fractures and widely used in previous finite element studies. Although our model was different from the other modeling studies, it was similar to the Zhang’s study [26], who divided the fracture into the following five fragments: anterior fragment, sustentaculum tali fragment, medial fragment, lateral fragment, and calcaneal tuberosity fragment. Considering that the sustentaculum tali might still be intact in a majority of fractures, we did not separate the medial fragments into two parts. For the implants model, we standardized the distribution and orientation of the screws on the plate in Techniques A and B. Three screws were applied for fixation of the medial and lateral fragment to stabilize the facet, with one screw aimed at the sustentaculum tali and two screws fixed at the anterior fragment. Three additional screws stabilized the tuberosity fragment. We added a medial support screw from the medial tuberosity to the medial fragment underneath the medial facet to enhance the stability of the medial column in Technique B. In Technique C, the fragments were stabilized by placing the screws in a triangular orientation. A lateral axial screw was inserted to restore the length of the calcaneal, while a medial screw was inserted for stabilization of tuberosity and to support the medial facet. Two additional screws were implanted to sustain the subtalar facet with one aimed at the sustentaculum tali.

Under physiological loading, we found that the maximum displacements of the fragments and implants were located at the sustentaculum tali and the tip of sustentaculum tali screw, respectively, which implied the relative weakness of the medial column and the need for a more stable fixation technique; however, Techniques B and C decreased the tendency for displacement of medial and lateral fragment, and the locking plate with a medial support screw fixation demonstrated a better result. We considered that with support for the medial column, the stability of the lateral fragment could increase subsequently, which would enhance the entire stability of the subtalar joint; therefore, we concluded that the medial support screw was necessary to augment the fixation of the facet. Similarly, the displacement of other fragments and implants was also less using Technique B than using the other two techniques and suggested that the fixation of a minimally invasive calcaneal locking plate with a medial support screw would provide better biomechanical strength for Sanders type II intra-articular calcaneal fractures.

Moreover, we found that Technique C yielded better support of the facet than the Technique A. We considered that the greater stability was achieved from the distribution of the screws. That is, three screws were inserted at different points for fixation of the subtalar joint, which included one screw in the axial plane to support the medial column. As the medial facet fragment was enhanced, another screw from the lateral facet to the medial facet subsequently provided greater stability, as previously mentioned; therefore, we concluded that the medial support was also critical for stabilizing the facet and that the medial screw plays an important role in this fixation method. We also suggested that regardless of which fixation technique is chosen, at least two screws should be applied to fix the subtalar facet to guarantee its stability.

From the maximum von Mises stress, we found that the stress of each fragment after using the three different fixation techniques was < 56 MPa, which indicated a low risk of screw loosening. In addition, the maximum von Mises stress of the implant after using the three techniques was less than its yield strength. We concluded that all of these techniques could achieve a stable fixation with less potential for implant breakage or failure. Although some difference in fragment stress existed among the three techniques, the difference in implants appears more obvious. In the present study, the maximum von Mises stress of the implants was the lowest in screw fixation. We suggested that the screw distribution within different dimensions and the intramedullary fixation style may play important roles in providing better implant stress dispersion. Furthermore, we found that with a medial support screw, the stress of the implants after using Technique B was less in an isolated locking plate model, which indicated that the medial support screw was also essential for implant stress dispersion.

## Study limitations

The present study suffered some limitations that should be considered when interpreting its findings, particularly when generalizing the findings to the clinical practice of general population. The main limitations of the study are as follows: (1) We simulated only the Sanders type II-B intra-articular calcaneal fracture using a simple model; therefore, it is not known whether our findings could be applied to other types of fractures, such as comminuted fractures. Therefore, to validate our findings, further controlled studies should be conducted; (2) evaluation of the biomechanical effects using the finite element analyses had some intrinsic limitations; therefore, we could not consider the findings as definite evidence for our conclusion, and the mechanical stability of the different fixation methods must be verified using classic biomechanical experiments; (3) any changes in model design, parameter settings, screw orientation, and implant position might affect the final result and cause bias; (4) in clinical work, screw distribution and orientation would be adjusted according to the fracture lines and the degree of comminution, which may also alter the strength of fixation and stress distribution; (5) we simplified the screw as a solid rod. However, the cannulated screws are more likely applied for classic screw fixation and medial support screw fixation in clinical practice. Whether the biomechanical stability could be compromised with cannulated screws still remains in doubt. Furthermore, the compression effect of cannulated screws could not be simulated in this model; and (6) although we set the same elastic modulus for plates and screws, in reality, differences exist, especially in different implant brands and designs. To resolve these limitations, conducting further well-designed and controlled studies in clinical setting as well as in modeling environments is necessary.

## Conclusion

All three of the minimally invasive fixation techniques could provide stable fixation for Sanders type II intra-articular calcaneal fractures; however, a medial support screw would provide greater stability, especially with a minimally invasive calcaneal locking plate fixation. Thus, the insertion of a medial support screw should be recommended in clinical practice to enhance the facet fragment. In addition, classic screw fixation could most effectively disperse stress, which is a reliable and economic method and should continue to be applied.
